# Emerging Therapeutic Options in Pancreatic Cancer Management

**DOI:** 10.3390/ijms25031929

**Published:** 2024-02-05

**Authors:** Donatella Delle Cave

**Affiliations:** Institute of Genetics and Biophysics ‘Adriano Buzzati-Traverso’, CNR, 80131 Naples, Italy; donatella.dellecave@igb.cnr.it

Pancreatic ductal adenocarcinoma (PDAC) is a devastating disease with a 5-year survival rate of <8% [1,2,3]. PDAC is characterized by dense desmoplastic stroma, which can constitute up to 90% of the tumor bulk, consisting of non-cellular and cellular components [4,5]. Pancreatic stellate cells (PSCs) and cancer-associated fibroblasts (CAFs) are the principal cellular components responsible for extracellular matrix (ECM) deposition and remodeling, and they play critical roles in cancer progression and treatment resistance [6]. PSC cells are present in a normal pancreas in a quiescent state and are principally involved in vitamin A storage within the cytoplasm [7]. After an injury, they activate and convert into a myofibroblast-like phenotype defined by α-SMA (alpha smooth muscle actin) expression, responsible for different ECM components’ secretion, in particular, fibronectins, laminins, and collagen, thus contributing to the highly fibrotic state of PDAC tumors [8,9,10]. CAFs in PDAC are most often divided into three subtypes, with different tumor-supporting capacities: myofibroblast-type CAFs (myCAFs), inflammatory CAFs (iCAFs), and antigen-presenting CAFs (apCAFs) [11,12,13]. Local approaches such as radiation therapy, hyperthermia, microwave or radiofrequency ablation, irreversible electroporation, and high-intensity focused ultrasound are capable of modifying the tumor microenvironment (TME) and ECM composition and structure, potentially enhancing chemotherapy [14]. Recently, immunotherapy has become a novel and promising alternative approach to target tumors [15,16,17]. The objective of immunotherapy is to (re)activate the immune system against cancer. Several innovative immunotherapies have been explored in pancreatic ductal adenocarcinoma (PDAC), with a focus on activating T cells either by blocking inhibitory signals or by enhancing their antitumor activity [18,19]. Stouten et al. focused their attention on the interactions between the immunosystem and CAFs, which can significantly influence immunotolerance and tumor growth [20]. The existing literature supports the role of CAFs in contributing to immunotherapy resistance; yet, the underlying mechanisms remain inadequately explored. Pathways associated with activated CAFs (iCAFs) are primarily considered pro-tumorigenic, although not exclusively. CAFs participate in desmoplasia, but the depletion of myCAFs can paradoxically lead to tumor progression. This presents a significant challenge for current and future therapeutic endeavors. At the moment, the combination of therapies targeting CAFs with immunotherapies has shown promising effects in murine models and certain clinical trials of PDAC patients [21,22]. PDAC typically progresses silently and without noticeable symptoms, leading to a challenging prognosis and poor outcomes [23,24]. Consequently, there is a pressing need to enhance early diagnosis and detect reliable biomarkers. In this context, one of the most promising approaches is the detection of exosomes in the bloodstream [25,26]. PDAC-associated microRNAs (miRNAs) packaged within exosomes could be used as diagnostic markers for the early detection of PDAC [27]. In this regard, Makler and colleagues identified four distinct differentially expressed miRNAs between plasma exosomes harvested from PDAC patients and those from control patients: miR-93-5p, miR-339-3p, miR-425-5p, and miR-425-3p, with an area under the curve (AUC) of the receiver operator characteristic curve (ROC) of 0.885, a sensitivity of 80%, and a specificity of 94.7%, which is comparable to the CA19-9 standard PDAC marker diagnostic [28]. Recently, the composition of the tumor microbiome has emerged as a novel prognostic factor for PDAC, as it differs from one patient to another and in response to chemotherapy [29,30,31,32]. Merali et al. demonstrated the existence of a bile microbiome signature in patients with PDAC who experienced obstructive jaundice caused by the disease, and the identification of specific bacteria in the bile has the potential to facilitate the detection and stratification of PDAC [33]. Deregulation of key signaling pathways in cancer, as well as altered genes expression, have critical functions in tumor progression [34,35,36,37,38]. Stukas et al. demonstrated that the inhibition of aryl hydrocarbon receptor (AHR) expression in PDAC sensitizes cells to gemcitabine, the gold standard treatment for pancreatic cancer, through the ELAVL1-DCK pathway [39,40,41,42,43]. Zuccolini et al. evaluated the effects of two different Ca^2+^-gated K^+^ channel KCa3.1 (commonly known as IK) blockers, namely Clotrimazole and Senicapoc, on metastatic melanoma and PDAC [44,45,46,47]. Although both inhibitors reduced the viability and migration of the tumor cells, neither of them altered the intracellular Ca^2+^ concentration in PDAC. In conclusion, the studies discussed in this editorial provide valuable insights into novel therapeutic strategies and personalized approaches for the treatment of PDAC. Further research in this field is needed to improve our knowledge regarding pancreatic cancer progression and to develop personalized treatments, with the aim of improving patient outcomes.
